# Recurrent Cytokine Release Syndrome in Metastatic Gastric Cancer on Nivolumab-FOLFOX: A Case Report and Mechanistic Review

**DOI:** 10.1007/s12029-025-01336-3

**Published:** 2025-11-04

**Authors:** Juned Islam, Zakaria Rashid, Alex B. Munster, Peta Hughes, Sasa Badzek, Inayah Huda

**Affiliations:** 1 Oncology Clinical Teaching Fellow at Whittington Hospital, Magdala Avenue, London, N19 5NF United Kingdom; 2 Honorary Clinical Lecturer (Teaching) at University College London, Gower St, London, WC1E 6BT United Kingdom; 3 Foundation Year Two Doctor in General Medicine at, Basildon University Hospital, Nether Mayne, Basildon, SS16 5NL United Kingdom; 4 Senior Clinical Fellow in Medical Oncology at Whittington Hospital, Magdala Avenue, London, N19 5NF United Kingdom; 5 Acute Oncology Service (AOS) Lead Nurse at Whittington Hospital, Magdala Avenue, London, N19 5NF United Kingdom; 6 Consultant in Medical Oncology at Whittington Hospital, Magdala Avenue, London, N19 5NF United Kingdom; 7 Clinical Pharmacist at Whittington Hospital, Magdala Avenue, London, N19 5NF United Kingdom; 8 General Pharmacy Practice Student at University College London, Gower St, London, WC1E 6BT United Kingdom

**Keywords:** Cytokine release syndrome, Gastric cancer, Nivolumab, Chemoimmunotherapy, Immune checkpoint inhibitors, Immune-related adverse events

## Abstract

**Introduction:**

Cytokine release syndrome (CRS) is a hyperinflammatory condition most commonly observed following chimeric antigen receptor (CAR)-T cell therapy in haematological malignancies. However, it remains rare and under-recognised in solid tumours.

**Case Presentation:**

We describe an 86-year-old woman with metastatic gastric adenocarcinoma treated with FOLFOX chemotherapy and nivolumab immunotherapy who developed recurrent episodes of CRS. Following the third and fourth treatment cycles, she presented with fever, confusion, abdominal pain, lymphadenopathy, leucocytosis, and acute kidney injury, with negative cultures and imaging. Both episodes resolved rapidly with supportive care alone. The recurrence of symptoms despite withholding granulocyte colony-stimulating factor (G-CSF) implicated chemoimmunotherapy as the likely trigger.

**Conclusion:**

This case highlights the diagnostic challenges posed by CRS, particularly its overlap with sepsis and other immune-related adverse events. We propose a dual mechanism in which cytotoxic chemotherapy amplifies immune checkpoint inhibitor-driven T-cell activation through tumour antigen release. Here, the reproducible treatment-linked pattern and resolution after stopping nivolumab supports an immune-mediated aetiology. Clinicians should maintain a high index of suspicion for CRS when evaluating gastric cancer patients presenting with fever and systemic inflammation after immunotherapy, as awareness is essential for timely recognition, differentiation from infection, and personalised management.

## Introduction

Gastric cancer remains a leading cause of cancer-related deaths worldwide [1]. While potentially curable at early stages, most patients present with advanced disease, which carries a poor prognosis [2]. Traditional first-line systemic therapies, typically comprising fluoropyrimidine and platinum-based regimens, have demonstrated limited survival benefit [3]. The emergence of immune checkpoint inhibitors (ICIs), particularly agents targeting the programmed death-1 (PD-1) pathway, has expanded treatment options for advanced gastric cancer. Nivolumab, in combination with FOLFOX chemotherapy, has shown improved overall survival in patients, as demonstrated in the CheckMate-649 trial [4], and is now an established first-line option.

However, ICIs are associated with a distinct spectrum of immune-related adverse events (irAEs), ranging from mild to life-threatening. Cytokine Release Syndrome (CRS), a hyperinflammatory state mediated by excessive cytokine production, is well characterised in patients receiving chimeric antigen receptor (CAR)-T cell therapy and bispecific antibodies, particularly in haematological malignancies [5, 6]. Yet, CRS remains rare and under-recognised in patients receiving ICIs for solid tumours [7, 27], with limited case reports and an uncertain incidence, often due to overlapping clinical features with infection or tumour-related inflammation. Here, we present a rare case of recurrent CRS in an 86-year-old patient with metastatic gastric adenocarcinoma treated with first-line nivolumab and FOLFOX. This report highlights the need for heightened clinical vigilance and early multidisciplinary intervention to mitigate the risks associated with rare but severe irAEs in the context of ICI-based therapies for solid tumours.

## Case Description

An 86-year-old woman, a retired nurse with mild frailty (Clinical Frailty Score of 4, and a performance status of 0), was diagnosed with stage IV poorly differentiated gastric adenocarcinoma with diffuse peritoneal disease since November 2024. Her medical history included well-controlled hypertension, type 2 diabetes mellitus, hypercholesterolaemia, asthma, stage 2 chronic kidney disease, diverticulosis, pre-diabetes, and a pulmonary embolism in December 2024, for which she received treatment-dose Tinzaparin injections. Her regular medications included Oramorph, Omeprazole, Amlodipine, Senna, Dihydrocodeine, Clenil modulite, and Dexamethasone on chemotherapy days; she denied any allergies. She commenced first-line palliative chemoimmunotherapy with FOLFOX (oxaliplatin, folinic acid, 5-fluorouracil) and the PD-1 inhibitor nivolumab in mid-January 2025. The first two cycles were well tolerated, with only mild toxicities like oral mucositis and fatigue. Neutropenia after the second cycle led to prophylactic granulocyte-colony stimulating factor (G-CSF) initiation from cycle 3 onwards.

### First Admission

Following her third cycle of 100% FOLFOX and nivolumab in mid-February, the patient presented with fever, confusion, jaw swelling (secondary to lymphadenopathy), and abdominal pain. These symptoms developed the day after G-CSF administration. Laboratory tests showed leucocytosis (elevated white cell count of 32.1 × 10^9^/L with high neutrophils of 30.5 × 109/L), elevated C-reactive protein (CRP) of 207 mg/L, and stage 1 acute kidney injury (AKI). CT imaging revealed colitis of uncertain aetiology, extensive gastric tumour necrosis, widespread skeletal metastasis, and lymphadenopathy. The presentation, characterised by systemic inflammation and Grade 2 neurological and gastrointestinal involvement without requiring vasopressors or high-flow oxygen, was consistent with Grade 2 Cytokine Release Syndrome (CRS) [8, 9]. She received empirical intravenous antibiotics and fluids for suspected neutropenic sepsis, with rapid clinical improvement within 24 h. Blood cultures, viral PCR, and imaging (including a chest x-ray and a CT Chest-Abdomen-Pelvis) found no infectious source. She was discharged within 48 h following stabilisation and normalisation of abnormal parameters.

### Between Admissions

In the weeks following discharge, she developed a symptomatic urinary tract infection (UTI), treated with oral nitrofurantoin. Chemotherapy was deferred by one week to allow for recovery.

### Second Admission

Cycle 4 of treatment was administered with a dose-reduced chemotherapy regimen (75% FOLFOX with 5-FU bolus omitted) and nivolumab in mid-March. The patient again presented with similar symptoms: fever, submandibular swelling, abdominal pain, fatigue, and shortness of breath, the day after treatment, but this time before G-CSF was administered. Laboratory findings revealed leucocytosis (WCC 31.9 × 10^9^/L, neutrophils 30.6 × 109/L), CRP 110 mg/L, lymphopenia, and stage 2 AKI, along with elevated liver enzymes. Microbiological investigations and imaging remained negative. The presentation, with fever, fluid-responsive hypotension, Grade 2 neurological (muddled, dazed), gastrointestinal, hepatic, and renal toxicities, was again consistent with Grade 2 CRS [8, 9]. Given the recurrent pattern, absence of G-CSF before symptom onset, and no identifiable infective source, immune-mediated CRS associated with nivolumab was deemed the most likely diagnosis. She was managed supportively with intravenous fluids and empirical antibiotics, and her chemotherapy pump was disconnected, again resulting in rapid clinical improvement and symptom resolution.

### Follow-Up

**Fig. 1:**
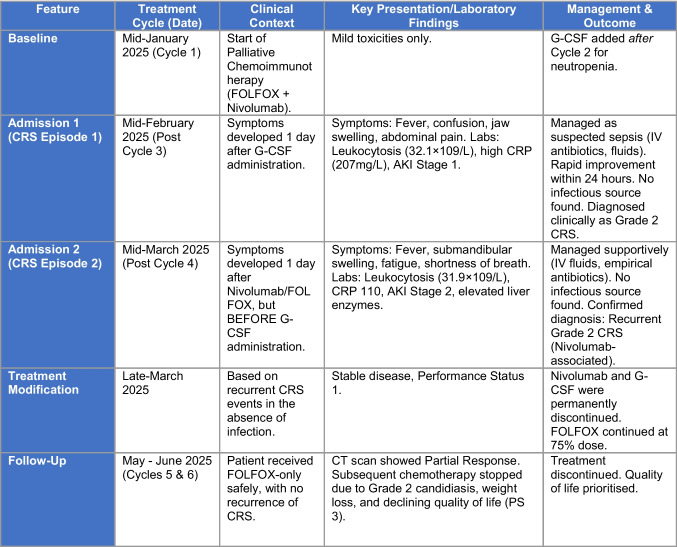
Timeline of Treatment, Presentations, and Management for Recurrent Cytokine Release Syndrome (CRS).The patient developed two distinct, reproducible episodes of Grade 2 CRS temporally linked to nivolumab administration. The second episode occurred *before* the administration of G-CSF, strengthening the diagnosis of immune checkpoint inhibitor-induced CRS and leading to the discontinuation of nivolumab. No further CRS episodes occurred following this

At a late-March outpatient oncology clinic review, the patient reported increased fatigue but no other symptoms, with a performance status of 1. A plan was made to administer FOLFOX at 75% without a 5-FU bolus from cycle 5, and nivolumab and G-CSF were removed from future prescriptions. Cycles 5 and 6 were safely administered in April with no complications. In early May, a CT scan revealed a partial response to treatment. Chemotherapy was planned for an additional three months, followed by reassessment. By mid-June, her condition significantly deteriorated due to recurrent oral and oesophageal candidiasis severely impacting her appetite, and substantial weight loss. Systemic antifungals were prescribed, chemotherapy was dose-reduced to 60%, and an endoscopy was arranged. Discussions about continuing chemotherapy balanced disease control with her declining performance status and quality of life. In late June, with ongoing Grade 2 candidiasis and high sepsis risk, the patient opted to discontinue chemotherapy. This decision was supported by the oncology team, prioritising quality of life given the persistent side effects, alongside a performance status of 3. A follow-up call was scheduled, after which she would be discharged from the clinic. Figure 1 provides a summarised timeline of key events and actions.

## Discussion

Cytokine release syndrome (CRS) is a well-characterised complication of cellular therapies, particularly chimeric antigen receptor T-cell (CAR-T) therapy, in haematological malignancies [6, 10]. It results from uncontrolled immune activation with excessive cytokine release, producing systemic inflammatory manifestations ranging from fever and malaise to shock and multi-organ dysfunction. Although once considered largely restricted to cellular therapies, growing evidence suggests that immune checkpoint inhibitors (ICIs) can also precipitate CRS in both haematological and solid tumours [7, 11]. Agents implicated include anti-programmed cell death protein 1 (PD-1) and anti-programmed death-ligand 1 (PD-L1) antibodies (e.g., nivolumab, pembrolizumab) as well as anti-cytotoxic T-lymphocyte antigen-4 (CTLA-4) antibodies (e.g., ipilimumab). However, the majority of cases arose from non-gastric cancers and detailed clinical characterisation was lacking. This report uniquely adds to the limited body of evidence describing recurrent CRS in metastatic gastric adenocarcinoma, a setting where the phenomenon remains under-recognised.

Compared with haematological malignancies, the incidence and severity of CRS in solid tumours appear lower. For example, an early trial of CAR-T in colorectal cancer and glioblastoma reported CRS in 34.6% of patients, though the majority were Grade 1–2 with only isolated severe cases [11]. Similarly, real-world pharmacovigilance data covering 2,672 patients with solid tumours, including gastric cancer, identified a 1.1% incidence of CRS, again mostly mild, but with occasional Grade ≥ 3 or fatal cases [12]. In contrast, CRS has been reported in 77–93% of patients with acute lymphoblastic leukaemia or diffuse large B-cell lymphoma receiving CAR-T, with up to 13% developing severe or life-threatening events [6, 13]. The gastric tumour microenvironment (TME) may partly explain the lower overall incidence: it is characterised by chronic inflammation, immunosuppressive stromal and immune cell populations, and antigen heterogeneity, all of which may dampen the systemic cytokine surge seen in haematological cancers where malignant cells are more directly accessible to T-cell–mediated killing [14–16]. These differences underscore the importance of documenting cases in gastric cancer to clarify patterns of presentation and risk.

A handful of case reports have linked ICIs to CRS in gastro-oesophageal malignancies, including nivolumab-associated events [17–19]. Other PD-1 inhibitors, such as tislelizumab, have also been implicated [20], and similar cases have been described in lung, cervical, and head and neck cancers [21–24]. This report adds important nuance by demonstrating recurrent CRS in metastatic gastric adenocarcinoma temporally associated with nivolumab–FOLFOX therapy. Unlike most published cases that describe a single CRS episode, our patient developed reproducible, treatment-linked episodes across consecutive cycles, providing rare support for causality. The reproducibility of symptoms in the absence of infection strongly supports an immune-driven mechanism and highlights the potential for cumulative immune sensitisation with continued ICI exposure.

Our case raises the possibility that chemotherapy may potentiate CRS risk by enhancing tumour antigen release, priming immune responses, and facilitating checkpoint inhibitor–mediated hyperinflammation. In this context, the combination of FOLFOX chemotherapy with nivolumab may have synergistically triggered the recurrent inflammatory cascades. Whereas CAR-T–associated CRS is typically driven by rapid T-cell proliferation and high levels of interleukin-6 and interferon-gamma [8, 25], chemotherapy-induced antigen release may act as an amplifier, precipitating delayed CRS. The timing here (after the third cycle) aligns with other reports of ICI-associated CRS, which often manifests later in treatment rather than at initiation [19, 27]. This pathophysiological model warrants further exploration, as ICIs are increasingly combined with cytotoxic backbones in gastrointestinal oncology.

Diagnosing CRS in solid tumour patients is particularly challenging because its manifestations overlap with neutropenic sepsis, tumour-related fever, and chemotherapy toxicity. In this case, the absence of an infectious source on microbiology and imaging, combined with rapid recovery after supportive care, supported an immune-mediated aetiology. The reproducibility of symptoms across successive treatment cycles further strengthens diagnostic certainty and suggests a priming effect, where repeated exposure heightened the inflammatory response. Development of novel diagnostic strategies, such as rapid cytokine profiling or cancer-specific clinical scoring systems, may allow earlier and more confident differentiation of CRS from sepsis in this population [26].

Whilst cytokine panels measuring IL-6, IFN-γ, and soluble IL-2 receptor levels were not used during this case, they may provide valuable diagnostic clues in similar cases, as elevations in these cytokines have been consistently observed in ICI- and CAR-T-associated CRS and correlate with severity [27–29]. IL-6 and ferritin have been reported to rise acutely in CRS compared with sepsis, aiding differentiation [30]. Conversely, infection biomarkers such as procalcitonin and C-reactive protein can help distinguish bacterial infection from immune-mediated inflammation: elevated procalcitonin supports the presence of bacterial infection rather than CRS, whereas CRP may rise in both contexts [31, 32]. Integrating these biomarkers into early assessment algorithms could improve diagnostic accuracy and guide timely intervention.

Clinicians should maintain a high index of suspicion for CRS in patients receiving ICIs, particularly when systemic symptoms such as fever, hypotension, or multi-organ dysfunction appear without an identifiable infectious source. Early steps include:

- Baseline and early cytokine monitoring (IL-6, IFN-γ, sIL-2R, ferritin) in high-risk patients or those presenting with atypical systemic inflammation [27–30].
- Concurrent infection workup, including procalcitonin, cultures, and imaging, to rule out bacterial causes [31, 32].
- Rapid multidisciplinary involvement, including oncology, microbiology, immunology, and intensive care, if systemic inflammatory features worsen [33].
- Supportive care, including fluid resuscitation, temporary treatment hold, and close monitoring of organ function [6, 10, 26].
- Corticosteroid therapy for recurrent or severe CRS, weighing risks of immunosuppression against benefits [6, 10, 34].
- Documentation and pharmacovigilance reporting to refine clinical understanding and guide future management strategies [35].

The potential contributory role of granulocyte colony-stimulating factor (G-CSF) should be acknowledged. In this case, CRS recurred despite the absence of G-CSF, suggesting a secondary rather than primary effect. Nevertheless, G-CSF has been associated with immune-related adverse events, including cytokine-driven phenomena, in other solid tumour contexts [36–38]. Its role should therefore not be dismissed and warrants further systematic investigation.

This case highlights the need for structured monitoring and management protocols for CRS in gastric cancer chemoimmunotherapy. Protocolised approaches incorporating dose modifications, corticosteroid prophylaxis in selected patients, or treatment discontinuation after recurrent CRS should be explored in prospective studies [6, 10, 34]. Expanding pharmacovigilance and registry data will be critical to refining evidence-based guidelines and improving patient safety in this emerging treatment landscape [35].

## Conclusion

This case highlights a rare but increasingly recognised immune-related complication of chemoimmunotherapy in solid tumours: cytokine release syndrome. In the context of metastatic gastric cancer, it underscores the diagnostic complexity and potential under-recognition of CRS, particularly when clinical features overlap with infection or chemotherapy toxicity. The reproducible symptom pattern, absence of infectious aetiology, and close temporal relationship to immunotherapy strongly support an immune-driven mechanism. As ICIs become more widely used in gastrointestinal malignancies, clinicians must remain vigilant for atypical irAEs like CRS, and exercise caution with adjuncts like G-CSF, which may confound diagnosis or exacerbate immune activation. Finally, they should tailor decisions on treatment continuation based on toxicity profile and overall clinical trajectory.

## Data Availability

No datasets were generated or analysed during the current study. Any relevant data to this patient case has already been included in the case report. If any further data is required, it is available on request from the corresponding author.
